# Surgery Treatment Improved the Overall Survival Rate in Locoregional Myxoid Leiomyosarcoma than Other Myxosarcomas in the United States

**DOI:** 10.1155/2021/9999529

**Published:** 2021-04-30

**Authors:** Marwan Almoiliqy, Abdullah Al-danakh, Mohammed Safi, Mohammed Alradhi, Mahmoud AL-Azab, Salah Adlat, Wanhai Zhou, Aiman Saleh A. Mohammed, Ahmed Al-maamari

**Affiliations:** ^1^Key Lab of Aromatic Plant Resources Exploitation and Utilization in Sichuan Higher Education, Yibin University, Yibin, 644000 Sichuan, China; ^2^Department of Pharmacology, Pharmaceutical College, Dalian Medical University, Dalian 116044, China; ^3^Department of Urology, First Affiliated Hospital of Dalian Medical University, Dalian 116044, China; ^4^Department of Oncology, First Affiliated Hospital of Dalian Medical University, Dalian 116044, China; ^5^Department of Urology, Second Affiliated Hospital of Dalian Medical University, Dalian 116044, China; ^6^Department of Immunology, Guangzhou Institute of Pediatrics, Guangzhou Women and Children's Medical Center, Guangzhou Medical University, Guangzhou 510623, China; ^7^Guangdong Cardiovascular Institute, Guangdong Provincial People's Hospital, Guangdong Academy of Medical Sciences, Guangzhou, Guangdong 510100, China; ^8^Department of Pharmacology and Pharmacotherapy, Faculty of Medicine, Interdisciplinary Excellence Center, University of Szeged, Szeged 6720, Hungary; ^9^Department of Pharmacology, School of Basic Medical Sciences, Xi'an Jiaotong University, Health Science Center, Xi'an 710061, China

## Abstract

Myxosarcomas are rare malignant tumors of soft connective tissues, classified into various subtypes, including myxoid liposarcoma, myxoid chondrosarcoma, and myxoid leiomyosarcoma. In this study, we proposed to study the demographic, tumor characteristics, and overall survival rate and compared the treatment modalities between these cancers. Patient data collected based on locoregional metastasis presentation of the abovementioned tumors with a cutoff study of survival duration up to 10 years were obtained from the SEER database during 1975-2016. Our results indicated that elderly patients and females were more in locoregional myxoid leiomyosarcoma than myxoid liposarcoma and myxoid chondrosarcoma with locoregional metastasis. The white race represented the most patients who suffered from these cancers than other races. The heart is the primary site for the abovementioned cancers, in addition to the female genitals to the myxoid leiomyosarcoma. Myxoid liposarcoma and myxoid chondrosarcoma patients with locoregional metastasis were suffering from grade II, while locoregional myxoid leiomyosarcoma patients with blank grading were due to missed data. Surgery was the most common treatment modality in this study compared with radiotherapy and chemotherapy. Kaplan-Meier analysis showed a significant difference in survival time between the three subtypes by using histology, and myxoid leiomyosarcoma showed prolonged survival than others. Elderly, female, white, unknown grade, surgery, no radiation, and no chemotherapy variables were independent factors associated with overall survival among these cancers. Multivariate analysis also showed significant differences in overall survival between the three tumors by histology, and myxoid leiomyosarcoma was with a better prognosis than others. Multivariate analysis of locoregional myxoid leiomyosarcoma showed the statistical significance of black race, grade, and radiotherapy, indicating them as independent prognostic factors of locoregional myxoid leiomyosarcoma. We conclude that surgery was the primary treatment modality against these cancers than radiotherapy and chemotherapy. And the locoregional myxoid leiomyosarcomas showed a better prognosis and higher survival rate than locoregional myxoid liposarcoma and locoregional myxoid chondrosarcoma.

## 1. Introduction

Myxosarcomas are rare malignant tumors of soft connective tissues [1]. Myxomatous areas indicated mucin deposition between the tumorous cells or in their exoplasm, including mesenchymal and epithelial cells [2]. Myxosarcoma mostly occurs in the heart [3, 4] but also may appear in other body locations including bone [5, 6], skin [7, 8], skeletal muscles [9], and ocular [10] and oral [11] cavities. Myxosarcomas are characterized histopathologically with stellate-shape and spare-spindle cells, neoplastic undifferentiated cells, mitotic figures, and mucoid stroma [12–14], and a low rate of myxosarcoma distant metastasis has been reported [15]. Myxosarcoma is mostly treated via surgery [16].

Myxosarcoma tumors are classified into various subtypes, including myxoid liposarcoma, myxoid chondrosarcoma, and myxoid leiomyosarcoma [17, 18]. Myxoid liposarcoma is a subtype of liposarcoma with a low risk of local recurrence than larger tumors [19]. Myxoid liposarcomas represent 5% of soft tissue sarcoma and 10-20% of liposarcoma [20]. However, myxoid liposarcomas may develop metastasis in almost 10% of myxoid liposarcoma patients and constitute 80% of the 5-year overall survival rate and 60% of the 10-year overall survival rate [21]. Myxoid chondrosarcomas are rare in extremities' soft tissues with less than 3% of soft tissue sarcomas [22]. Despite the myxoid chondrosarcomas characterized with a high risk of local recurrence and high spread rate of metastasis, several studies reported a prolonged survival rate of almost 70% of 10-year overall survival [22–24]. Myxoid leiomyosarcomas are a subtype of leiomyosarcomas characterized as rare, aggressive, and well-recognized uterus tumors [25]. Myxoid leiomyosarcoma showed a poor prognosis and a worse overall survival rate of 5 years, almost 11% [26].

Directed locoregional therapy on the metastasis pattern with additional treatment of the primary tumors may reduce the primary tumor burden, reduce metastasis prognosis, and improve the overall survival rate than systemic treatment to the distant metastasis [27–29]. It is reported that primary tumor resection could promote distant metastasis [30, 31] while this hypothesis was drawn by recent studies and elucidated clinically the role of surgery in reducing the distant metastasis and improving the overall survival rate [32, 33].

The Surveillance, Epidemiology, and End Results (SEER) is a comprehensive program of incidence, prognosis, and cancer surveillance in the United States (U.S.). SEER is an authoritative program that provides information on cancer statistics in the U.S. to minimize the cancer prognosis on the U.S. population. SEER is supported by the Surveillance Research Program (SRP) in the National Cancer Institute's (NCI's) Division of Cancer Control and Population Sciences (DCCPS). Among 19 registries around the U.S., cancer data has been collected through a coordinated system. These geographic registries reflect about 35% of the U.S. population, which represents the entire U.S. demographics. Data collection started with a few registries in 1973 and expanded to include more areas in the U.S. [34–36].

Myxoid liposarcomas, myxoid chondrosarcomas, and myxoid leiomyosarcomas are rare tumors with low incidence and survival data, so a comparison study has also been challenging to clarify. So, we proposed to study the demographic, tumor characteristics, and overall survival rates and to compare the treatment modalities between locoregional metastatic myxoid liposarcomas, locoregional metastatic myxoid chondrosarcomas, and locoregional metastatic myxoid leiomyosarcomas during 1975-2017 in 18 registries of the United States based on SEER database analysis.

## 2. Methods

### 2.1. Data Collection

#### 2.1.1. Study Cohort

Patient data of myxoid liposarcoma, myxoid chondrosarcoma, and myxoid leiomyosarcoma were obtained from the SEER database of cases diagnosed during 1975-2016 in 18 registries of the United States [37]. All patients were identified based on the international classification of disease for oncology, third edition (ICD-O-3), using codes of myxoid liposarcoma (8850-8889), myxoid chondrosarcoma (9180-9249), and myxoid leiomyosarcoma (8890-8929). The data extracted from the SEER database in this study was based only on the locoregional metastasis presentation of myxoid liposarcoma, myxoid chondrosarcoma, and myxoid leiomyosarcoma tumors. We obtained a total number of patients of about 1398 of locoregional myxoid liposarcoma, 356 of locoregional myxoid chondrosarcoma, and 129 of locoregional myxoid leiomyosarcoma. Cases with unknown or missing data are included in the descriptive and survival analysis.

#### 2.1.2. Study Measurement

This study's primary measurement is to identify the locoregional metastasis differences of demographic, tumor characteristics, and survival rates between these three rare myxoid tumors (myxoid liposarcoma, myxoid chondrosarcoma, and myxoid leiomyosarcoma) among 18 registries of the United States during1975-2016. All variables included in this study are based on the cutoff study of survival duration up to 10 years [37].

#### 2.1.3. Variable Study

All patient data are extracted from the SEER database. Demographic data were organized as age at diagnosis, gender, and race. The age variable was studied in two categories (less than 50 and greater than 50) to ease the analysis. The race was categorized as white, black, and others (Asian and others). The anatomical locations were categorized into six variables, including soft tissue including heart, retroperitoneum, bones and joints, female genitals, digestive organs, and others. Oncology study includes grading and survival analysis. The tumor grade in the SEER database was classified into grades I, II, III, IV, and unknown. Overall survival rate analysis is performed based on the study cutoff selection of patient data from SEER. So, all cases in this study considered only the patients with 10-year survival (120 months). The SEER database's treatment variables, including surgery, radiation, and chemotherapy, were included in this study. In surgery treatment, the patients were categorized into two variables: whether they performed the surgery or not (yes/no). Radiation and chemotherapy variables include the patients who received treatment or not (yes/no) [37].

### 2.2. Statistical Analysis

Data analysis was performed using SPSS software version 22 (SPSS, IBM Company, Chicago, USA). Chi-squared tests are used to analyze the variables. Categorical variables were analyzed as frequency and percentage. Ten-year overall survival was calculated using Kaplan-Meier to estimate the survival rate with a log-rank test. Multivariate Cox proportional hazard regression was studied to analyze the confounding variables. *p* < 0.05 was used as a significant value in the study.

## 3. Results

### 3.1. Myxoid Liposarcoma

#### 3.1.1. Demographics

Among 1398 locoregional metastatic patients of myxoid liposarcoma extracted from the SEER database during 1975-2016, almost 49.1% of cases with age less than 50 years and 50.9% of cases with age greater than 50 years were diagnosed with locoregional myxoid liposarcoma. Myxoid liposarcomas were higher in males than females, with almost 59.4% and 40.6% of cases, respectively. The race variable reported that the white race of almost 82.5% was higher than black and other races of almost 9.7% and 7.9%, respectively, in this tumor, indicating that the male gender and white people were more vulnerable to this cancer. The location of the myxoid liposarcoma tumors showed higher localization of cancer in the heart, which is almost 91.5% of the reported cases compared with other sites (Table 1).

#### 3.1.2. Oncology Characteristics

The reported data of the locoregional metastatic patients during 1975-2016 and obtained from the SEER database with a total number of 1398 myxoid liposarcomas in the United States showed that this tumor has high grading II (32.8%) versus grade I (27.9%), grades III-IV (7.6%-8.0%), and unknown (23.7%) indicating that locoregional metastatic patients with myxoid liposarcomas have high grading of this tumor. The cause of death of most cases of myxoid liposarcoma was not attributed to this tumor as reported in SEER data with almost 91.4% (Table 1).

#### 3.1.3. Treatment

Locoregional metastatic myxoid liposarcoma treatment data are extracted from SEER during 1975-2016, including surgery, radiotherapy, and chemotherapy modalities. Most of the reported cases in the SEER of locoregional metastatic patients with myxoid liposarcoma were performed surgery which is almost 95.2% of the total number of patients, and this result was similar as in the literature [38]. Radiotherapy treatments of patients with locoregional myxoid liposarcoma of data obtained from SEER showed no big difference between received beam radiations versus unreceived patients, almost 47.1% versus 52.9%. Meanwhile, chemotherapy variables showed a higher data of patients who did not receive chemotherapy which is about 89.5% compared with the patients who received treatment which is about 10.5%, and these results of treatment modality indicate that surgery treatment modality was the most performed treatment approach in locoregional myxoid liposarcoma patients (Table 1).

### 3.2. Myxoid Chondrosarcoma

#### 3.2.1. Demographics

The data of locoregional metastatic patients with myxoid chondrosarcoma obtained from SEER were 356 patients. There are almost 55.1% of cases with age less than 50 years versus 44.9% of cases with age greater than 50 years. The sex variable of locoregional metastatic myxoid chondrosarcomas reported that the male variable was higher than the female variable with almost 59.8% and 40.2% of cases, respectively. White patients were also higher than black and other races, about 76.7%, 14.6%, and 8.7%, respectively, in this tumor, indicating that this tumor mostly affects male and white people than others. The localization of the locoregional metastatic myxoid chondrosarcoma tumor was higher in the heart and bones/joints, almost 64.0% and 32.3%, respectively, of the reported cases compared with other sites (Table 1).

#### 3.2.2. Oncology Characteristics

The data obtained from the SEER database of locoregional metastatic patients with myxoid chondrosarcomas showed that this tumor has higher grade II (32.6%) and unknown (38.8%) versus grades I, III, and IV (13.8%, 9.8%, and 5.1%, respectively), indicating that locoregional metastatic patients with myxoid chondrosarcomas exhibit high grading of this tumor. The cause of death of most cases of locoregional metastatic myxoid chondrosarcomas also was not attributed to this tumor as reported in SEER data with almost 76.7% (Table 1).

#### 3.2.3. Treatment

Most of the cases obtained from the SEER database with locoregional metastatic myxoid chondrosarcomas were performed surgery with almost 89% of the patients' total number. And data obtained of locoregional metastatic patients with myxoid chondrosarcomas did not show a big difference between cases that received radiotherapy or not, almost 44.1% and 55.9%, respectively. The chemotherapy variables showed a higher data of patients who did not receive chemotherapy, about 82.9%, compared with the patients who received treatment which is almost 17.1%, indicating that surgery is the primary treatment modality in locoregional metastatic myxoid chondrosarcoma patients than radiation and chemotherapy (Table 1).

### 3.3. Myxoid Leiomyosarcoma

#### 3.3.1. Demographics

Myxoid leiomyosarcomas with locoregional metastatic patients were obtained from the SEER database in the U.S. during 1975-2016, almost 129 cases. The age variable showed that about 63.6% of cases diagnosed with locoregional myxoid leiomyosarcoma were with age greater than 50 years versus 36.4% of cases with age less than 50 years, indicating that this tumor mostly occurs in the older population. The sex variable of locoregional myxoid leiomyosarcoma patients reported that the female variable was higher than the male variable with almost 82.2% and 17.8% of cases. White patients were higher than black and other races, almost 77.5%, 18.6%, and 3.9%, respectively, in this tumor, indicating that the female and white people were more vulnerable to this cancer. The myxoid leiomyosarcoma tumors were higher localized in the female genitals and heart, almost 57.4% and 33.3%, respectively, of the reported cases compared with other sites (Table 1).

#### 3.3.2. Oncology Characteristics

The tumor grading variable of locoregional myxoid leiomyosarcoma reported cases showed that this tumor has higher unknown grading (40.3%) versus grades II, III, and IV (16.3%, 19.4%, and 16.3%, respectively) and less grade I (7.8%). The cause of death of locoregional myxoid leiomyosarcoma cases of almost 72.1% was not attributed to this tumor as reported in SEER data (Table 1).

#### 3.3.3. Treatment

Most of the locoregional myxoid leiomyosarcoma patient's data obtained from the SEER database are performed surgery with almost 91.5% of the total number of patients. And the data of patients with locoregional myxoid leiomyosarcoma extracted by SEER did not show a big difference between groups receiving and not receiving radiotherapy of almost 45.7% and 54.3%, respectively. And most of the cases did not receive chemotherapy treatments, about 86% in this tumor (Table 1).

### 3.4. Survival Analysis

**Figure 1:**
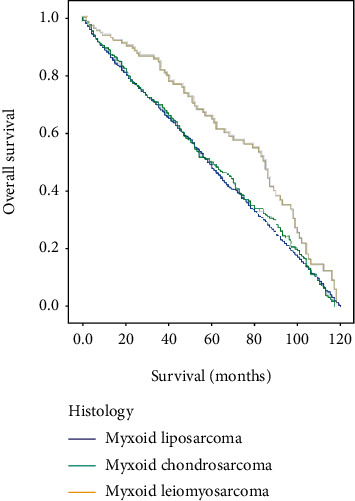
Overall survival of patients with locoregional myxoid liposarcoma, locoregional myxoid chondrosarcoma, and locoregional myxoid leiomyosarcoma by histology via Kaplan-Meier analysis with log-rank test and median 58 months, 60 months, and 85 months, respectively, *p* < 0.004.

**Figure 2:**
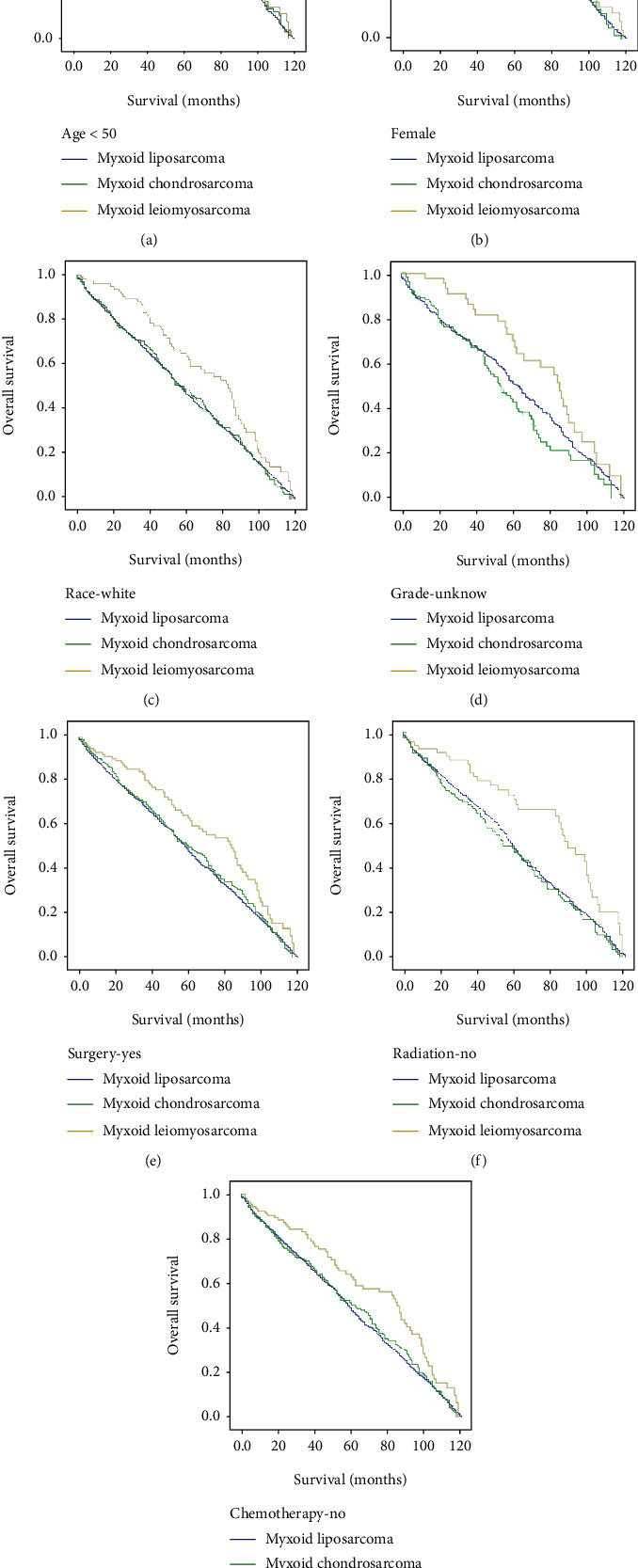
Overall survival of patients with locoregional myxoid liposarcoma, locoregional myxoid chondrosarcoma, and locoregional myxoid leiomyosarcoma via Kaplan-Meier analysis with log-rank test: (a) age more than 50 years, *p* < 0.008; (b) female gender, *p* < 0.007; (c) white race, *p* < 0.014; (d) unknown grade, *p* < 0.017; (e) surgery resection, *p* < 0.008; (f) radiation (no), *p* < 0.002; (g) chemotherapy (no), *p* < 0.009.

**Figure 3:**
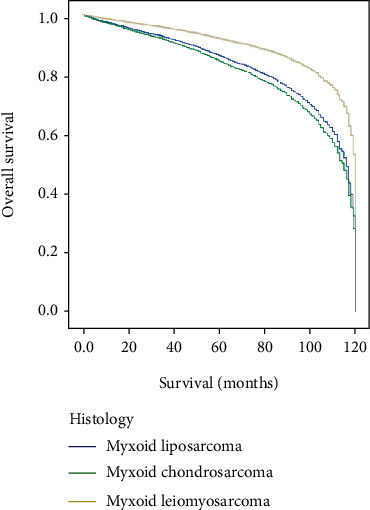
Multivariate analysis of patients with locoregional myxoid liposarcoma, locoregional myxoid chondrosarcoma, and locoregional myxoid leiomyosarcoma using Cox hazard proportion study.

Kaplan-Meier analysis of overall survival showed a statistically significant difference between histology subtypes of myxoid liposarcoma, myxoid chondrosarcoma, and myxoid leiomyosarcoma of locoregional metastatic patients with log-rank *p* < 0.004 (Figure 1). And locoregional metastatic myxoid leiomyosarcoma patients showed prolonged survival (median 85 months) compared with locoregional metastatic myxoid liposarcoma (median 58 months) and locoregional metastatic myxoid chondrosarcoma (median 60 months) (Figure 1). The Kaplan-Meier study indicated that variables of age more than 50 years, female gender, white race, unknown or missed grade, surgery resection, no radiation, and no chemotherapy variables showed statistical significance of overall survival (Table 2). Patients older than 50 showed statistical significance of survival with a median of 58 months of locoregional myxoid liposarcoma, 63 months of locoregional myxoid chondrosarcoma, and 85 months of locoregional myxoid leiomyosarcoma with log-rank *p* < 0.008 (Table 2, Figure 2), indicating that patients with locoregional myxoid leiomyosarcoma showed prolonged survival than others. Female patients of locoregional myxoid leiomyosarcoma with a median of 85 months showed significantly prolonged survival time compared with locoregional myxoid liposarcoma and locoregional myxoid chondrosarcoma (61 months and 54 months), *p* < 0.007 (Table 2, Figure 2). And white patients also showed prolonged survival in locoregional myxoid leiomyosarcoma (83 months) compared with locoregional myxoid liposarcoma and locoregional myxoid chondrosarcoma (58 months and 59 months, respectively), *p* < 0.014 (Table 2, Figure 2). The tumor grade showed that unknown or missed grading in myxoid leiomyosarcoma has significantly higher overall survival (median 85 months) compared with other tumors (63 and 54 median months, respectively), suggesting the inadequate data collection and poor follow-up of patients to their registries, *p* < 0.017 (Table 2, Figure 2). Among the treatment modalities, surgery resection of myxoid leiomyosarcoma tumors in locoregional metastatic presentation results in prolonged survival time with a median of 84 months compared with locoregional myxoid liposarcoma and locoregional myxoid chondrosarcoma, *p* < 0.008 (Table 2, Figure 2). The locoregional myxoid leiomyosarcoma patients who were not receiving radiotherapy and chemotherapy showed a significant difference in overall survival time and lived prolonged than other locoregional myxoid liposarcoma patients and locoregional myxoid chondrosarcoma patients with log-rank *p* < 0.002 and *p* < 0.009, respectively (Table 2, Figure 2). Multivariate analysis of histology subtype was also meaningful that showed a statistically significant difference of overall survival between myxoid liposarcoma, myxoid chondrosarcoma, and myxoid leiomyosarcoma of locoregional metastatic patients, *p* < 0.0001, and the locoregional myxoid leiomyosarcoma cases reported better survival time than other cancers (Figure 3). Based on the abovementioned results and comparing these three tumors, we selected the locoregional myxoid leiomyosarcoma to perform univariate and multivariate analyses. And the univariate analysis of locoregional myxoid leiomyosarcoma showed no statistically significant difference in overall survival (Table 3). Moreover, multivariate analysis showed that black race, with a hazard ratio (0.363), 95% CI (0.154-0.855), *p* < 0.021; grade IV with a hazard ratio (3.748), 95% CI (1.234-11.384), *p* < 0.02; and radiotherapy with a hazard ratio (0.529), 95% CI (0.302-0.926), *p* < 0.026 were independent prognostic factors for locoregional myxoid leiomyosarcoma patients according to data obtained from SEER (Table 3).

## 4. Discussion

Myxosarcomas are malignant soft tissue tumors characterized by abundant mucus [39]. Myxosarcoma was classified into several subtypes, including myxoid liposarcoma, myxoid chondrosarcoma, and myxoid leiomyosarcoma [18]. Metastasis presentation of myxoid liposarcomas, myxoid chondrosarcomas, and myxoid leiomyosarcomas was higher with locoregional metastasis in all three abovementioned cancers compared with distant metastasis indicating the rare distant metastasis in these cancers, interpreting that locoregional metastasis may provide a prolonged overall survival rate than distant metastasis [40–43]. The SEER database is an authorized software providing statistical information on cancers among several registries in the U.S. [34]. This study performed statistical analysis of demographic, tumor study, and survival among patients of locoregional myxoid liposarcoma, locoregional myxoid chondrosarcoma, and locoregional myxoid leiomyosarcoma in the U.S. based on data extracted from the SEER database during 1975-2016 [37].

Our demographic analysis of locoregional myxoid leiomyosarcomas showed that most patients were older than 50 compared with locoregional myxoid liposarcomas, and locoregional myxoid chondrosarcoma patients were showing no big difference between age variables according to data reported by SEER, and these results are similar to that reported in literature revealing that myxoid leiomyosarcomas occurred mostly in the elderly [44–46]. Myxoid liposarcoma and myxoid chondrosarcoma tumors with locoregional metastasis have mostly male predilection [44, 47, 48], and the locoregional myxoid leiomyosarcoma tumor has commonly female predilection [49]. Our obtained data indicated that cases of myxoid liposarcomas and myxoid chondrosarcomas with locoregional metastasis were mostly male patients. In contrast, locoregional myxoid leiomyosarcoma patients were mainly female due to myxoid leiomyosarcoma's histological origin, which commonly grows in the uterus [50]. White patients were the most patients suffering from the abovementioned cancers compared with other races, indicating that the white race is more vulnerable to these cancers than other races, as reported in several studies [51, 52]. Our data were indicating that the white race represented the most patients suffering from these cancers in the U.S., according to the SEER database. Myxosarcoma tumors can be grown in various body locations but mostly grow in the heart [53, 54]. In this study, our analysis indicated that locoregional myxoid liposarcoma and locoregional myxoid chondrosarcoma tumors were primarily located in the heart and locoregional myxoid leiomyosarcoma tumors are located mostly in female genitals and heart too.

Oncology analysis of our data obtained from SEER showed that locoregional myxoid liposarcoma and locoregional myxoid chondrosarcoma patients suffering from tumors have grade II differentiation. In contrast, locoregional myxoid leiomyosarcoma patients with blank or unknown grading showed a higher percentage compared with another grading, suggesting that the higher unknown cases in locoregional myxoid leiomyosarcomas were due to the less number of patient data collection and poor follow-up to their registries during that time of registration, which provides missing data compared with other well-reported cancers; however, grades II, III, and IV were higher in locoregional myxoid leiomyosarcoma compared with grade I.

Surgery is the most common approach of treatment modality used against myxoid liposarcomas, myxoid chondrosarcomas, and myxoid leiomyosarcomas compared to radiotherapy and chemotherapy [55, 56]; interestingly, our analysis in this study indicated that the most reported cases of myxoid liposarcomas, myxoid chondrosarcomas, and myxoid leiomyosarcomas with locoregional metastasis were treated with surgery. Meanwhile, in radiotherapy modality, our analysis did not show any statistical significance among myxoid liposarcomas, myxoid chondrosarcomas, and myxoid leiomyosarcomas with locoregional metastasis. And chemotherapy treatment against locoregional metastasis of myxoid liposarcomas, myxoid chondrosarcomas, and myxoid leiomyosarcomas showed statistical significance with a higher percentage of patients who did not receive chemotherapy.

The overall survival rate was better for locoregional localization than distant metastasis, as reported in previous studies [27, 57, 58]. Our study's overall survival based on Kaplan-Meier analysis showed a statistically significant difference in survival time between patients with locoregional metastasis of myxoid liposarcomas, myxoid chondrosarcomas, and myxoid leiomyosarcomas by using the histology variable. The locoregional metastasis of myxoid leiomyosarcoma showed prolonged survival than locoregional metastatic myxoid liposarcoma and locoregional metastatic myxoid chondrosarcoma. Kaplan-Meier analysis indicated that age older than 50 years, female gender, white patients, unknown or missed grade, surgery, no radiation, and no chemotherapy variables were independent factors associated with overall survival among locoregional metastasis of myxoid liposarcomas, myxoid chondrosarcomas, and myxoid leiomyosarcomas. As reported in the literature, myxoid leiomyosarcomas are associated with a worse prognosis and low overall survival rate [26]; surprisingly, our data extracted from the SEER database of locoregional metastatic myxoid leiomyosarcomas reflected a better prognosis and high survival rate compared with locoregional metastatic myxoid liposarcomas and locoregional metastatic myxoid chondrosarcomas, suggesting that locoregional metastasis is accompanied with prolonged survival and a better prognosis than distant metastasis. Meaningfully, multivariate analysis using Cox hazard proportion analysis between the three subtypes of myxoid liposarcomas, myxoid chondrosarcomas, and myxoid leiomyosarcomas with locoregional metastasis showed also a significant difference in the overall survival rate. However, the locoregional myxoid leiomyosarcoma showed a better survival rate than other abovementioned subtypes, so the univariate and multivariate analyses of locoregional myxoid leiomyosarcoma had been performed, and the results of the univariate study did not show statistical significance of overall survival. The multivariate analysis showed the statistical significance of the black race, tumor grade, and radiotherapy variables of locoregional myxoid leiomyosarcoma, indicating that variables were independent prognostic factors of locoregional myxoid leiomyosarcoma based on the collected data from the SEER database. By comparing the overall survival of three subtypes of locoregional myxoid liposarcomas, locoregional myxoid chondrosarcomas, and locoregional myxoid leiomyosarcomas, the results illustrated that locoregional myxoid leiomyosarcomas have better prognostic and overall survival than the other subtypes.

In conclusion, in this study, we analyzed the demographic, oncology, and survival patterns of myxoid liposarcoma, myxoid chondrosarcoma, and myxoid leiomyosarcoma locoregional metastasis in the U.S. during 1975-2016 based on the SEER database. Our results were similar to the literature, indicating that white patients suffered more from myxoid liposarcoma, myxoid chondrosarcoma, and myxoid leiomyosarcoma with locoregional metastasis. The heart was the most common organ to develop locoregional metastasis of myxoid liposarcoma, myxoid chondrosarcoma, and myxoid leiomyosarcoma associated with high-grade differentiations. And surgery was the main approach of treatment modality against these cancers than radiotherapy and chemotherapy. The survival patterns showed that elderly, female, white patients, unknown or missed grade, surgery, radiation, and chemotherapy were dependent factors of overall survival among these cancers. However, locoregional myxoid leiomyosarcomas showed a better prognosis and better survival rate than locoregional myxoid liposarcoma and locoregional myxoid chondrosarcoma based on Kaplan-Meier and Cox hazard proportion studies. Adjuvant radiation and chemotherapy against locoregional leiomyosarcoma based on multicenter randomized clinical trials need further study in the future.

## Figures and Tables

**Table 1:** Demographic data, tumor characteristics, and treatment modality of locoregional myxoid liposarcoma, locoregional myxoid chondrosarcoma, and locoregional myxoid leiomyosarcoma in the Unites States based on the SEER database.

Demographic chch.	Myxoid liposarcoma Total (*n* = 1398) (%)	Myxoid chondrosarcoma Total (*n* = 356) (%)	Myxoid leiomyosarcoma Total (*n* = 129) (%)	*p* value sig (0.05)
Disease				
Age (years)				0.001
<50	687 49.1%	196 55.1%	47 36.4%	
>50	711 50.9%	160 44.9%	82 63.6%	
Sex				0.0001
Male	831 59.4%	213 59.8%	23 17.8%	
Female	567 40.6%	143 40.2%	106 82.2%	
Race				0.002
White	1153 82.5%	273 76.7%	100 77.5%	
Black	135 9.7%	52 14.6%	24 18.6%	
Other	110 7.9%	31 8.7%	5 3.9%	
Grade				0.0001
Grade I	390 27.9%	49 13.8%	10 7.8%	
Grade II	459 32.8%	116 32.6%	21 16.3%	
Grade III	106 7.6%	35 9.8%	25 19.4%	
Grade IV	112 8.0%	18 5.1%	21 16.3%	
Unknown	331 23.7%	138 48.8%	52 40.3%	
Primary site				0.0001
Heart	1279 91.5%	228 64.0%	43 33.3%	
Retroperitoneum	68 4.9%	1 0.3%	5 3.9%	
Bones and joints	4 0.3%	115 32.3%	0 0.0%	
Female genital organs	3 0.2%	0 0.0%	74 57.4%	
Digestive system	10 0.7%	0 0.0%	4 3.1%	
Others	34 2.4%	12 3.4%	3 2.3%	
Surgery				0.0001
Yes	1331 95.2%	317 89.0%	118 91.5%	
No	67 4.8%	39 11.0%	11 8.5%	
Radiotherapy				0.596
Yes	658 47.1%	157 44.1%	59 45.7%	
No	740 52.9%	119 55.9%	70 54.3%	
Chemotherapy				0.002
Yes	147 10.5%	61 17.1%	18 14.0%	
No	1251 89.5%	259 82.9%	111 86.0%	
Cause-specific death to cancer				0.0001
Yes	120 8.6%	83 23.3%	36 27.9%	
No	1278 91.4%	273 76.7%	93 72.1%	

chch: characteristics.

**Table 2:** Overall survival study using Kaplan-Meier analysis on locoregional myxoid liposarcoma, locoregional myxoid chondrosarcoma, and locoregional myxoid leiomyosarcoma in the United States.

Demographic chch	Myxoid liposarcoma Total (*n* = 1389)	Myxoid chondrosarcoma Total (*n* = 356)	Myxoid leiomyosarcoma Total (*n* = 129)	Log-rank sig (0.05)
Disease				
Age (years)				
<50	59	54	79	0.073
>50	58	63	85	0.048
Sex				
Male	57	69	85	0.227
Female	61	54	85	0.007
Race				
White	58	59	83	0.014
Black	62	67	102	0.318
Other	61	71	—	0.538
Grade				
Grade I	55	47	98	0.361
Grade II	59	70	70	0.226
Grade III	56	75	83	0.195
Grade IV	60	92	62	0.610
Unknown	63	54	85	0.017
Surgery				
Yes	59	62	84	0.008
No	49	45	85	0.411
Radiotherapy				
Yes	38	47	54	0.268
No	59	54	89	0.002
Chemotherapy				
Yes	56	49	79	0.431
No	59	62	85	0.009

chch: characteristics.

**Table 3:** Univariate and multivariate analyses on locoregional myxoid leiomyosarcoma in the United States.

Demographic chch	Myxoid leiomyosarcoma Total (*n* = 129)					
Disease						
	Univariate			Multivariate		
	Hazard ratio	95% CI (lower-upper)	*p* value	Hazard ratio	95% CI (lower-upper)	*p* value
Age (years)						
<50 *vs.* >50	1.054	0.655-1.699	0.827	0.619	0.336-1.14	0.124
Sex						
Male *vs.* female	0.887	0.493-1.597	0.690	1.731	0.618-4.848	0.296
Race						
White	Ref			Ref		
Black	0.764	0.399-1.462	0.416	0.363	0.154-0.855	0.021
Other	0.613	0.149-2.518	0.498	1.555	0.358-6.756	0.556
Grade						
Grade I	Ref			Ref		
Grade II	1.149	0.449-2.939	0.772	1.406	0.519-3.803	0.503
Grade III	0.901	0.360-2.256	0.824	1.068	0.410-2.786	0.892
Grade IV	1.195	0.468-3.053	0.710	3.748	1.234-11.384	0.02
Unknown	0.933	0.405-2.148	0.870	1.130	0.475-2.686	0.783
Surgery						
Yes *vs.* no	0.881	0.320-2.425	0.806	1.431	0.360-5.681	0.611
Radiotherapy						
Yes *vs.* no	1.593	0.993-2.557	0.053	0.529	0.302-0.926	0.026
Chemotherapy						
Yes *vs.* no	0.971	0.435-2.169	0.943	0.740	0.292-1.873	0.525

chch: characteristics; ref: reference.

## Data Availability

The data used in this study are available in the Surveillance, Epidemiology, and End Results (SEER) database of the National Cancer Institute (http://seer.cancer.gov) and available with the corresponding author.

## Abbreviations

SEER: Surveillance, Epidemiology, and End Results
U.S: United States
SRP: Surveillance Research Program
NCI: National Cancer Institute
DCCPS: Division of Cancer Control and Population Sciences
ICD-O-3: International classification of disease for oncology third edition.
